# Development and validation of an antibiotic appropriateness metric for urinary tract infections and pyelonephritis in ambulatory settings

**DOI:** 10.1017/ash.2024.490

**Published:** 2025-01-17

**Authors:** Mackenzie R. Keintz, Bryan T. Alexander, Katherine Fagan, Trevor C. Van Schooneveld

**Affiliations:** 1 Division of Infectious Diseases, University of Nebraska Medical Center, Omaha, NE, USA; 2 Department of Pharmaceutical & Nutrition Care, Nebraska Medicine, Omaha, NE, USA; 3 Analytics Team, Nebraska Medicine, Omaha, NE, USA

## Abstract

Ambulatory antibiotic stewardship has traditionally focused on acute respiratory infections with few studies evaluating metrics for other commonly encountered ambulatory conditions, including urinary tract infections (UTI). We describe the development and validation of an electronically captured appropriate antibiotic use metric for ambulatory UTIs using coding data.

## Introduction

Antimicrobial resistance is a significant problem in the United States^1^. The key driver of antimicrobial resistance is antibiotic use, making judicious antibiotic prescribing essential. Most antibiotics are prescribed in the ambulatory care setting^2^. Ambulatory antibiotic prescribing remains high with an estimated 709 antibiotic prescriptions per 1,000 persons in 2022^3^. Ambulatory antibiotic stewardship has traditionally focused on acute respiratory infections (ARI), with little focus on other common infectious conditions that generate antibiotic prescriptions. Urinary tract infections (UTI) account for about 10% of all antibiotics prescribed in the ambulatory care setting with an estimated 70% of those being suboptimal^4,5^. No accepted ambulatory metric exists for evaluating antibiotic use for UTI. We developed and validated a metric to assess appropriate antibiotic prescribing for UTI in a network of 17 primary and immediate care clinics associated with an academic medical center.

## Methods

### Identifying the patient population

We utilized ICD-10 codes to identify ambulatory visit encounters for UTI at 13 primary care and four immediate care clinics at 1 academic health system. Diagnostic codes included acute uncomplicated UTI, complicated UTI including pyelonephritis, and asymptomatic bacteriuria to ensure capture of all antibiotic use for genitourinary infection. We excluded patients with ICD-10 codes for pregnancy, renal transplant, neutropenia, nephrolithiasis, immunosuppression, urinary catheter use, or codes that would warrant an antibiotic for other reasons (Supplement 1). Additionally, we excluded patients with antibiotic duration >28 days or with an exclusionary urologic procedure.

### Information extracted from the electronic medical record (EMR)

Patient characteristics including age, gender, race, and ethnicity were extracted from the EMR. We collected visit information including clinic, provider, diagnosis code, antimicrobial agent, dose, frequency, and duration.

### Defining appropriateness

Appropriate use metrics were created within the 3 defined indications: uncomplicated UTI, complicated UTI, and asymptomatic bacteriuria. For a UTI antibiotic prescription to be defined as appropriate, it needed to be either a first- or second-line agent, utilize the correct total daily dose, and be given for the correct duration (Table 1). We utilized SQL Server Management Studio to categorize antibiotic prescription orders from the EMR as appropriate or inappropriate based on the defined variables. We also created individual component sub-metrics for appropriate agent choice, dose, and duration.

**Table 1.** Appropriate antimicrobial agent, dose, frequency, and duration for uncomplicated urinary tract infection

Antimicrobial	Dose^a^	Frequency^a^	Duration
**First-line antibiotics**			
Nitrofurantoin	100 mg	2 times daily	5 d
Trimethoprim-sulfamethoxazole	160/800 mg	2 times daily	3 d
Fosfomycin	3 g	Once	–
**Second-line antibiotics**			
Cephalexin	500–1000 mg	2–3 times daily	5–7 d
Amoxicillin	500–1000 mg	2–3 times daily	5–7 d
Amoxicillin/clavulanate	875/125 mg	2 times daily	5–7 d
Cefadroxil	500–1000 mg	2 times daily	5–7 d
Cefuroxime	250 mg	2 times daily	5–7 d
Cefdinir	300 mg	2 times daily	5–7 d
Cefpodoxime	100 mg	2 times daily	5–7 d
**Not routinely recommended**			
Levofloxacin	–	–	–
Ciprofloxacin	–	–	–
Moxifloxacin	–	–	–
Doxycycline	–	–	–
Minocycline	–	–	–
Omadacycline	–	–	–
Azithromycin	–	–	–
Clindamycin	–	–	–
Metronidazole	–	–	–

a Dose and frequency listed for normal renal function; appropriateness was assessed considering proper patient-specific renal adjustments, as necessary.

### Validating the metric

To verify the electronic metric accurately identified, classified, and categorized outcomes, we evaluated 6 months of data (October 1, 2023–March 31, 2024) using manual chart review of both identified and excluded cases. First, patient and visit data pulled from the EMR were compared to data obtained via chart review. We also determined whether the adjudication performed by the software followed the appropriate rules. Finally, clinical documentation was reviewed to determine if encounter coding was accurate, as well as if there was a variance in prescribing due to clinical factors the metric was unable to include, such as culture data, renal function, or allergy.

## Results

During the validation period, 575 clinic visits were coded for uncomplicated UTI, pyelonephritis, or asymptomatic bacteriuria. Most visits captured were secondary to uncomplicated UTI (98%, 566 of 575) (Table 2). In 572 antibiotic prescriptions written, the algorithm correctly identified all variables assessed. Two prescriptions for a diagnosis other than UTI were inappropriately included, and 1 antibiotic duration was incorrectly captured (Table 2). All antibiotic prescriptions were correctly adjudicated as appropriate or inappropriate based on set variables for overall appropriateness and the individual components (agent, dose, and duration).

**Table 2.** Patient and visit characteristics and metric performance

Characteristic	All diagnoses	Uncomplicated UTI % (n)	Complicated UTI % (n)	Asymptomatic bacteriuria % (n)
	–	98 (566/575)	1 (8/575)	0.1 (1/575)
**Gender**				
Male	11 (61/575)	10 (59/566)	13 (1/8)	100 (1/1)
Female	89 (514/575)	90 (507/566)	87 (7/8)	(0/1)
**Age**				
18–50	40 (230/575)	39 (223/566)	75 (6/8)	100 (1/1)
51–64	24 (138/575)	24 (138/566)	(0/8)	(0/1)
>65	36 (207/575)	36 (205/566)	25 (2/8)	(0/1)
**Visit setting**				
Primary care clinic	55 (315/575)	54 (304/566)	63 (5/8)	100 (1/1)
Immediate care clinic	45 (265/575)	46 (262/566)	37 (3/8)	(0/1)
**Metric accuracy**				
Diagnostic code	99.7 (573/575)	–	–	–
Overall appropriate antibiotic		99.8 (565/566)	100 (8/8)	100 (1/1)
Appropriate agent		100 (566/566)	100 (8/8)	100 (1/1)
Appropriate duration		99.8 (565/566)	100 (8/8)	100 (1/1)
Appropriate dosage		100 (566/566)	100 (8/8)	100 (1/1)
**Inappropriately coded**	14 (80/575)	14 (78/566)	13 (1/8)	100 (1/1)
Appropriate code uncomplicated UTI		–	13 (1/8)	(0/1)
Appropriate code complicated UTI		27 (29/78)	–	100 (1/1)
Appropriate code ASB or none		63 (49/78)	(0/8)	–

Note. UTI, urinary tract infection; ASB, asymptomatic bacteriuria.

After evaluating clinical documentation, 80 clinic visits were inappropriately coded (78 uncomplicated UTI, 1 complicated UTI, 1 asymptomatic bacteriuria) (Table 2). Forty-nine of 78 (63%) incorrectly coded uncomplicated UTI visits resulted from no documented symptoms. Complicated UTI should have been coded in 29 of 78 (37%) uncomplicated-coded visits (Table 2). Most patients (411 of 575 [71%]) had a urine culture sent. Only 13 of 411 (3%) cultures revealed a pathogen where at least one first- or second-line antibiotic was inactive.

## Discussion

Ambulatory stewardship interventions that utilize tracking and reporting of appropriate antibiotic use with feedback have improved antibiotic use in ambulatory ARI^6^. Adapting this strategy to UTI requires developing a valid metric as the first step. Treatment metrics for UTI with feedback have been deployed in emergency departments as a part of a multicomponent strategy with some success^7^. To our knowledge, this is the first validated metric for appropriate antibiotic use in UTIs developed for the ambulatory care setting.

Accurate measures are essential, and our metric was highly effective at identifying and adjudicating appropriate antibiotic use for UTIs in the ambulatory care setting. We did identify errors in the coding of visits that may result in inappropriate adjudication. Most complicated UTI visits were coded as uncomplicated, which could affect metric performance. The most common error was a lack of symptom documentation in patients coded as uncomplicated. This may be due to either inadequate clinical documentation or inaccurate coding. It does highlight an issue with the metric in that it is not able to evaluate clinical documentation, and therefore, inappropriate treatment of asymptomatic bacteriuria that is coded as a UTI would be adjudicated as appropriate. Coding errors are a recognized issue when utilizing this data to determine appropriateness and can be mitigated by educating providers on metric scoring methodologies and encouraging coding to match the clinical diagnosis, as well as documentation of any potential complicating factors that may result in deviation or exclusion^8^.

We reviewed culture data as this can influence prescribing and may be an appropriate reason to not utilize a first- or second-line agent. Culture rates were exceedingly high for what would often be considered uncomplicated UTI, but it allowed us to compare this data to our metric. The rate of visits where culture data would dictate a non-first- or second-line agent was very low, highlighting the utility of this metric. It is worth noting that clinical situations do exist (eg, allergy, previous culture data, reduced renal function), which would warrant the use of alternative agents or dosing considered “inappropriate” per the metric. Analysis is underway to inform at what level this metric should be set. Setting a goal of 100% “appropriateness” would not be in line with good clinical care, and metrics such as ours must be calibrated to account for exceptions and still be able to identify areas for clinical improvement.

Limitations of our study include implementation within a single health system. Our metric used ICD-10 coding data as opposed to clinical diagnosis, with our validation demonstrating that 13% of visits were miscoded. Part of developing metrics based on coded diagnoses is ensuring appropriate coding data are provided by clinicians and ensuring accurate coding will be a target for our intervention. Additionally, this metric may have missed antibiotic prescriptions for UTI if no code for genitourinary syndrome was entered. This metric only captures a portion of UTI visits, as we chose not to include those in which another infectious condition or complications (stones, catheter, etc.) were present. The inclusion of these populations would make an automated metric exceedingly challenging.

We validated our electronic metric measuring appropriate UTIs in the ambulatory care setting, which accurately identifies UTI cases and adjudicates antibiotic use. We plan to implement provider- and clinic-level metric evaluation as part of a quality improvement initiative in our clinics, with clinician feedback. This may be a useful tool for other institutions as it can be implemented to monitor and presumptively improve appropriate antibiotic prescribing.

## Supporting information

Keintz et al. supplementary material Keintz et al. supplementary material

## Supplementary material

For supplementary material accompanying this paper visit http://doi.org/10.1017/ash.2024.490. click here to view supplementary material
