# Supplementary material for: Development and validation of an antibiotic appropriateness metric for urinary tract infections and pyelonephritis in ambulatory settings
Source: Antimicrob Steward Healthc Epidemiol. 2025 Jan 17;5(1):e8. doi: 10.1017/ash.2024.490 (PMC11748013; doi:10.1017/ash.2024.490)
Supplement: Keintz et al. supplementary material [file S2732494X2400490Xsup001.docx]

| **Inclusion ICD-10 codes** |  |
| --- | --- |
| Uncomplicated cystitis | N39.0 |
|  | N30.00 |
|  | N30.01 |
|  | N30.80 |
|  | N30.81 |
|  | N30.90 |
|  | N30.91 |
|  | N34.1 |
|  | N34.2 |
|  | N34.3 |
|  | N39.9 |
|  | Z87.440 |
| Complicated cystitis | N10 |
| Asymptomatic bacteriuria | R82.71 |
| **Exclusion ICD-10 codes** |  |
| Pregnancy | Z34.90 |
|  | Z3A.01 |
|  | Z3A.08-Z3A.42 |
|  | Z3A.49 |
|  | O48.1 |
|  | O98.911-O98.919 |
|  | Z33.1 |
|  | Z32.01 |
|  | O23.40 |
|  | B95.5 |
|  | O23.41 |
|  | O99.891 |
| Renal Transplant | T86.10 |
|  | Z94.0 |
| Neutropenia | D70.9 |
|  | D70.1-D70.4 |
|  | Z91.89 |
| Nephrolithiasis | N20.0 |
| Immunosuppression | D84.9 |
|  | D84.821 |
| Urinary Catheter Use | T83.9XXA |
|  | Z96.00000 |
|  | T83.518A,S,D |
|  | Z46.6 |
|  | T83.511A,S,D |
|  | T83.098A,S,D |
|  | Z91.89 |
| Additional code requiring antibiotics | H05.01* |
|  | H60.0* |
|  | H60.1* |
|  | H60.2* |
|  | H60.3* |
|  | H60.6* |
|  | H60.8* |
|  | H60.9* |
|  | H62.4* |
|  | H66.00* |
|  | H66.01* |
|  | H66.9* |
|  | H67.* |
|  | H70.00* |
|  | H70.01* |
|  | H70.09* |
|  | H70.9* |
|  | H79.89* |
|  | J01.* |
|  | J02.* |
|  | J03.* |
|  | J04.3* |
|  | J05.* |
|  | J15.* |
|  | J18.* except J18.2 |
|  | K57.0* |
|  | K57.2* |
|  | K57.4* |
|  | K57.8* |
|  | K61.* |
|  | K65.* except K65.3 & K65.4 |
|  | L01.* except L01.02 & L01.03 |
|  | L02.* |
|  | L03.01* |
|  | L03.03* |
|  | L03.11* |
|  | L03.31* |
|  | L03.81* |

Supplement 1. Inclusion and exclusion ICD-10 codes.
